# Graphene nanoplatelets on recycled rubber: an experimental study of material properties and mechanical improvements

**DOI:** 10.1098/rsta.2023.0324

**Published:** 2024-10-23

**Authors:** J. M. Londono Monsalve, E. Kovalska, M. F. Craciun, M. R. Marsico

**Affiliations:** ^1^ Department of Engineering, University of Exeter, Exeter EX4 4QF, UK

**Keywords:** recycled rubber, graphene-reinforced polymers, cyclic testing, Raman, FTIR

## Abstract

This study presents an experimental investigation of the mechanical behaviour of recycled rubber pads coated with graphene nanoplatelets. The investigation is part of an effort to develop a novel rubber-based composite that aims to reroute rubber from end-of-life tyres from illegal landfills and incineration back into the market in the form of a novel composite for vibration isolation. Graphene nanoplatelets were deposited on rubber pads via ultrasonic spray coating. The pads were made of a combination of recycled rubber (from tyres) and virgin rubber. A comprehensive analysis of the structural and chemical properties of the graphene coating, ensuring its integrity on the rubber substrate, was performed by combining surface topography, Raman and Fourier-transform infrared (FTIR) spectroscopy. Stacked coated pads were cured and tested dynamically in compression and shear under cyclic loading. Results showed promising improvements in the mechanical properties, in particular, in compressive stiffness and damping of the coated specimens with respect to their uncoated counterparts, laying the foundation for using graphene-enhanced recycled rubber as a novel composite.

This article is part of the theme issue ‘Celebrating the 15th anniversary of the Royal Society Newton International Fellowship’.

## 1. Introduction

The rubber industry is under increasing pressure to adopt more sustainable practices, not only including responsible rubber production and ethical supply chains, but also closing the loop through rubber recycling [1]. Recycling rubber from tyres is crucial, as annually, over 25 million tonnes (Mt) of tyres reach the end of their useful lives [2]. End-of-life tyres (ELTs) are not reusable tyres that are legally classified as non-hazardous waste. The recycling industry has developed technologies for recovering the rubber from ELT, nonetheless, the lack of demand in applications incorporating ELT-recycled rubber into products constitutes a lasting and significant obstacle in ELT management (about 55 million tyres/year in the UK [3]). In the EU alone, this results in more than 1 Mt of ELT devaluated annually, so being incinerated (in energy recovery processes) with the environmental cost of about 700 kg of CO^_2_^ emissions per tonne [4]. This paper delves into the promising avenue of incorporating graphene into recycled rubber to enhance its mechanical properties, thus enabling its use in engineering applications (e.g. elastomeric bearings). Researchers have increasingly focused on nanomaterials to enhance the mechanical properties of rubber and other elastomers [5]. Several studies have investigated the effects of filling elastomers with two-dimensional materials, for instance, Sadasivuni *et al*. [6] explored the properties of various graphitic fillers incorporated into an elastomeric matrix, observing that graphene, due to its exceptional mechanical and electrical properties, stands out as a promising nanofiller for reinforcement. The best reinforcement has been obtained in elastomeric nanocomposites with efficient graphene dispersion [7] and strong graphene–polymer interfaces [8]. Several researchers have highlighted the important role of the formation of filler networks in the matrix [9–11] and small particle sizes of filler [8,12,13] in improving the overall composite material performance. Graphene was also found to benefit the rubber vulcanization kinetics [11], reducing curing times [14] and enhancing the vulcanization rate [15]. However, despite the promising benefits of adding graphene to rubber, achieving a uniform and stable dispersion of graphene particles in a rubber matrix remains a challenge. While researchers continue to investigate ways to improve graphene dispersion [16–19], to the best of the authors’ knowledge, there is still no process to disperse graphene on rubber that can combine high efficiency, low cost and environmental safety [20].

Layered composites are an alternative approach for reinforcing rubber with graphene. The approach of spraying graphene onto rubber enables precise control of graphene layering, resulting in a more uniform distribution of this nanomaterial within the polymer matrix, and producing composites with improved mechanical properties [21,22]. Spray coating is preferred here due to its high material utilization, low manufacture costs and compatibility to streamline production [23]. Additionally, the environmental footprint of the spraying approach is reduced compared with other graphene deposition methods, such as chemical vapour deposition [24], and it is scalable suggesting a potential for more cost-effective production methods [25]. Nonetheless, further research is still needed to optimize the graphene concentration to strike the right balance between mechanical reinforcement and material cost-effectiveness.

The interest here is to discuss our observations on the mechanical behaviour of a material that we believe represents an alternative approach to reinforcing reclaimed rubber with graphene, a new composite made with recycled rubber pads interposed with a few layers of graphene, which is deposited using electrostatic adhesion [26]. In this paper, we investigate an experimental study of this novel material which holds promise for energy absorption and vibration reduction. These two pivotal aspects have been central to Dr J. M. Londono Monsalve’s project on ‘Enhanced structural control systems for damage reduction during earthquakes’, which was funded by the prestigious Newton International Fellowship awarded to him in 2010. In what follows, we first describe the method used to disperse and deposit the GNPs onto the recycled rubber (RR) pads. Following this and to appreciate the chemical composition of the rubber pads on which we have deposited a few layers of graphene, we discuss the results obtained using the Raman spectroscopy and Fourier-transform infrared spectroscopy (FTIR). Additionally, we discuss the results of a microscopic analysis conducted to trace the profile and the topography of the rubber pads coated with graphene nanoplatelets, so as to assess the uniformity of the graphene coating. Finally, we present the results of the mechanical tests (compression and shear tests) performed on scaled-down specimens, that were manufactured by stacking up a few graphene-coated pads and curing them in a hot press machine.

## 2. Material preparation

In this study, we have used a rubber compound consisting of 50% virgin rubber and 50% ELT–RR mix (handmade in the form of 3.5-mm-thick pads) which were coated with graphene nanoplatelets (GNPs). The preparation of graphene dispersion in deionized water was performed by processing it with a high-shear mixer (Silverson lab mixer, L5SU model, a 32  mm diameter rotor head) at a rotor speed of 5000 rpm for 120 min. The graphene dispersion with a concentration of 1.25 g l^−1^ was achieved. Subsequently, the dispersion was subjected to 15 min of ultrasonication before being deposited onto the rubber substrate. The deposition of graphene onto the rubber pads was conducted using an automated programmable benchtop ultrasonic spray coater with a 300 × 300 mm work area. The spray coater is equipped with an ultrasonic nozzle, which effectively (re)disperses graphene particles in water and promotes a uniform wide-area coating of graphene on the rubber pads. The deposition procedure was carried out at low-pressure air (6.5  bar) and a low flow rate of 0.7  ml min^−1^. The spray coater is also equipped with a hot plate where the rubber substrates are placed. To ensure a high-quality coating, the hot plate temperature was set at 140°C, rapidly evaporating the water-based solvent and promoting the proper interaction of fine graphene nanoplatelets with the rubber surface.

To deposit graphene on rubber pads, we have investigated the use of brass masks with thicknesses of 0.06 and 0.2 mm, respectively. Results obtained with the 0.06-mm-thick brass mask were unsatisfactory as it was found to be too thin. Although the mask was secured to the substrate on the outer edges, it fluttered under the flow of the spray coater, resulting in the deposition of graphene nanoplatelets on areas designated to be uncoated. The stiffer 0.2-mm-thick brass mask was employed for all specimens discussed in this study since it was discovered that if manually pressed onto the rubber substrate while heating it, it would adhere to it for the entire duration of the spray coating.

For the morphology and chemical characterization (§3), three pads were prepared: pad 1 was made with uncoated and not pre-heated rubber substrate, pad 2 was made by depositing 150 cumulative cycles of graphene nanoplatelets on the rubber substrate and pad 3 was made by depositing 150 cumulative cycles of graphene nanoplatelets on the rubber substrate drop cast in a hydrophobic solution (patent application number PCT/GB2023/051786) to enhance adhesion during vulcanization. The specimens were coated using a 0.2-mm-thick brass mask with a distinct pattern designed where the perimetral region is left uncoated to facilitate the adhesion between stacked coated pads. Table 1 presents a summary of the prepared specimens.

**Table 1.** Specimens for morphology and chemical characterization.

specimen	coating cycles	drop casted
pad 1	—	no
pad 2	150	no
pad 3	150	yes

For the mechanical characterization (§4), we manufactured several specimens of the composite by stacking several 20 × 20 mm graphene-coated RR pads and curing them in a manually operated hydraulic laboratory hot press. The coated RR pads were confined in a purpose-built steel mould during curing. Control specimens were also manufactured using uncoated RR pads. To facilitate data comparability, all the specimens were manufactured at the same temperature, pressure and duration of the curing period. After curing, the specimens have a final thickness of 15 mm. Adherence among layers has been assessed qualitatively by visually inspecting coated and uncoated specimens cut in half after curing, with curing temperature and time have been adjusted to ensure layer bonding. Adherence has also been confirmed through the deformed shape of the specimen during the shear test as shown in figure 10. In this test, since the composite is layered parallel to the plates’ direction, one would expect the layers to slide with respect to each other in the absence of bonding, thus, the fact that the specimens deform as a solid block implies layer bonding.

The tested specimens for the mechanical characterization are summarized in table 2. Specimens E35–E36 were made with four pad 3 type; a patterned mask was used to deposit graphene nanoplatelets covering 16% of the surface area. Specimens E39–E40 were made with four pad 2 type and 40% coated area; an additional layer, uncoated not pre-heated was positioned on top with a thickness of about 1.5 mm to match the height of the other specimens (compensating the unevenness of the handmade pads). Control specimens E43–E44 were made with four uncoated pads pre-heated (matching the thermal exposure of coated pads on the spray coater’s hot plate); once again, adding on top a not pre-heated and uncoated rubber pad 1.5-mm thick.

**Table 2.** Specimens tested in compression and shear.

specimens	recycled to natural rubber content	pad thickness (mm)	coating cycles	% coated area
E35–E36	50%	3.5	150	16
E39–E40	50%	3.5	150	40
E43–E44	50%	3.5	—	—

## 3. Morphology and chemical characterization

To analyse graphene coating on rubber pads, we used a complementary approach combining Raman and FTIR spectroscopy to provide a multi-faceted understanding of the material’s properties. Surface topography analysis and scanning electron microscopy analysis were utilized to supplement the morphological dimensions of the graphene coatings by examining their texture, uniformity and roughness.

### (a) Raman spectroscopy

Raman spectroscopy stands as the foremost method when it comes to characterizing carbon materials such as graphene [27,28]; it offers detailed structural information, such as layers number, quality and defects presence, while also identifying stress and strain due to interaction between graphene and rubber substrate. Raman test serves as a fast, non-destructive, high-resolution tool for studying lattice structure and a variety of properties, including electronic, optical and phonon attributes.

Raman spectroscopy was performed on different locations on the rubber pads as summarized in table 3, with three measurements taken at each location. In particular, on pad 1 location: RR1, on pad 2 location: G-RR2 graphene-coated area and location RR3 uncoated area, and on pad 3 locations: f-RR5 uncoated area and f-G-RR4 graphene-coated area.

**Table 3.** Locations for Raman and FTIR spectroscopic characterization.

specimen	graphene-coated location	uncoated location
pad 1	—	RR1
pad 2	G-RR2	RR3
pad 3	f-G-RR4	f-RR5

**Figure 1.**
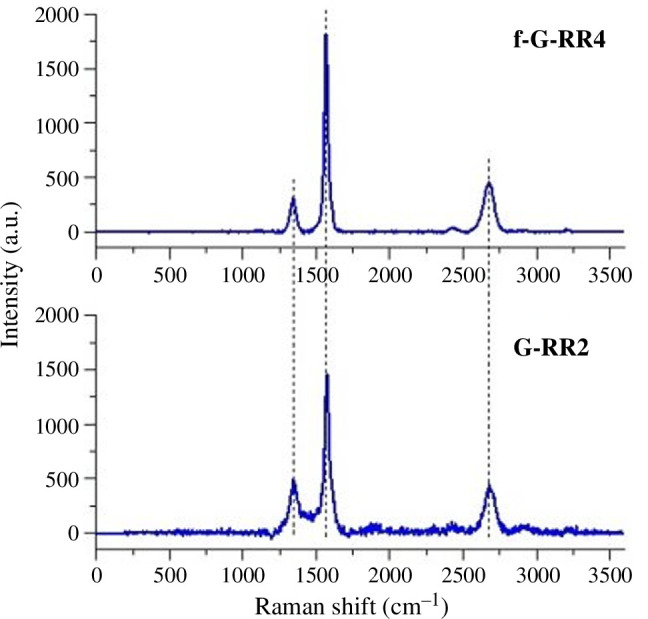
Raman spectra of f-G-RR4 (f-RR5 subtracted) graphene-coated and drop cast recycled rubber, and of G-RR2 (RR3 subtracted) graphene-coated recycled rubber. The *y*-axis is the intensity of the scattered light represented in arbitrary units (a.u.). The *x*-axis is the Raman shift that refers to the energy (frequency) of light measured in cm^−1^.

Raman analysis was employed here to assess the quality of graphene layers on rubber substrates before and after functionalization, utilizing a 532-nm laser, 600 g mm^−1^ grating, 50× objective, and 50 × 0.5 s integration time. The Raman spectra from G-RR2 and f-G-RR4 exhibited typical profiles of multilayer graphene, showing prominent G- and two-dimensional peaks at 1559 and 2676 cm^−1^, respectively (figure 1). The presence of a low-intensity D-band (1348 cm^−1^) indicated intrinsic structural defects in graphene nanoplatelets, with RR2 exhibiting a higher intensity D-band due to increased defectiveness compared with f-G-RR4. Notably, Raman analysis suggested that the quality of the graphene-coated area remained consistent and unaffected by the hydrophobic coating in both samples.

### (b) Fourier-transform infrared spectroscopy

**Figure 2.**
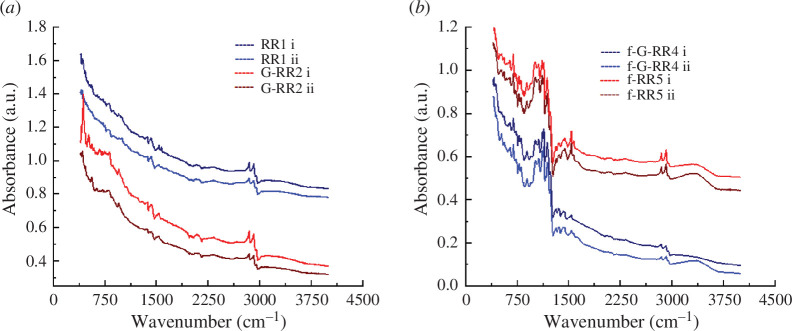
FTIR spectra (scans i and ii) showcasing: (*a*) recycled rubber (RR1) and graphene-coated recycled rubber (g-rr2); (*b*) the comparison between RR and G-RR after their functionalization with hydrophobic coating (f-RR5 and f-G-RR4, respectively). The *x*-axis represents the wavenumber (cm^−1^) and the *y*-axis represents the absorbance of infrared light in arbitrary units (a.u.).

To enhance our understanding of chemical interactions and modifications at the graphene–rubber interfaces, we performed a Fourier-transform infrared (FTIR) spectroscopy as an analytical method employed for the identification of functional groups (organic, polymeric or inorganic) and chemical bonds of surfaces [29]. In this study, the FTIR spectra at locations RR1, G-RR2, f-G-RR4 and f-RR5 were acquired to assess the chemical composition of their surfaces. The measurements were conducted employing infrared (IR) spectroscopic imaging technology, which allows for the simultaneous measurement of all light frequencies [30]. The FTIR spectra, repeated twice for each location (denoted as i and ii), are showcased in figure 2*a,b* depicting both uncoated (RR) and graphene-coated (G-RR) areas of recycled rubber pads as well as their functionalized forms. Analysis of the non-functionalized samples in figure 2*a* confirms the presence of similar polymeric units in both the G-RR and RR regions of the pads [30]. Further analysis of the FTIR spectra reveals the –CH_3_ asymmetric stretching at 2961 cm^−1^ for RR, while the –CH_2_ symmetric stretching vibrations are observed in the range of 2846–2916 cm^−1^ for the G-RR sample, indicating the successful deposition of sp^2^-bonded carbon atoms (graphene coating) onto the rubber. The peaks at 1370, 1424, 1538 and 1574 cm^−1^ are attributed to –CH_2_ deformation, which may be influenced by the heating of the samples during the spray coating process, potentially impacting their elastic properties. In the case of functionalized samples (figure 2*b*), we observe a distinctive set of peaks at 1000–1300 cm^−1^, corresponding to the -CF stretching. This feature arises from the application of a hydrophobic coating on the surface of pad 3 (f-RR4 and f-G-RR5), intentionally employed to bolster adhesion between them. The vibrations of –CH_3_ and –CH_2_ stretching are also present in the range of 2000–2200 cm^−1^; however, they appear quenched by the hydrophobic coating, resulting in lower intensities compared with the non-functionalized samples. Additionally, a broad peak around 3374 cm^−1^ is indicative of methyl and methylene group vibrations, as well as –OH stretching vibrations, which emerge following the sample surface functionalization. The absorption rate remains consistent across all samples and analysed areas, indicating that there is no observable direct impact of surface functionalization (attributed to hydrophobic coating) and temperature (resulting from heating during graphene spraying) on the structural properties of the rubber.

### (c) Surface topography

**Figure 3.**
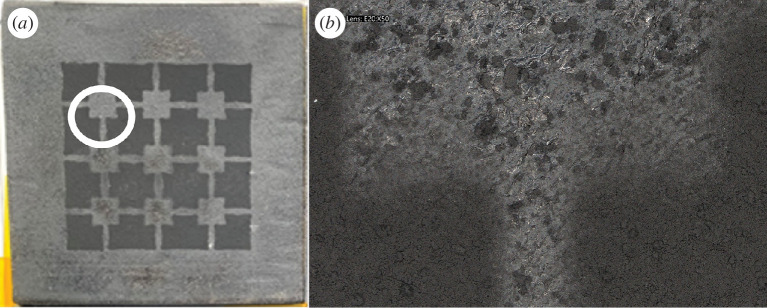
(*a*) Image of the specimen with 150 cumulative cycles of graphene nanoplatelets. Patterned graphene coating; (*b*) lens E20 × 50.

The investigation on the surface topography and cross-sectional profiles was conducted using a VHX 7000 Digital Microscope Keyence. Figure 3*a* shows a general view of a patterned graphene-coated pad while figure 3*b* shows a 50× magnification of a small region on it. It can be observed that the boundaries between coated and uncoated areas are not always defined consistently and look blurry. We believe this is due to the method used for the deposition of the graphene nanoplatelets; as wherever the patterned brass mask did not adhere perfectly to the rubber substrate, it was unable to accommodate the deformation of the substrate when heated at 140°C. We noticed that both the rubber substrate and brass mask bend under heating during the spray coating, but they neither do it at the same rate nor follow an identical bending configuration due to the cut-outs in the mask. This likely resulted in graphene nanoplatelets penetrating underneath the mask edges, making the boundary between coated and uncoated areas blurred.

**Figure 4.**
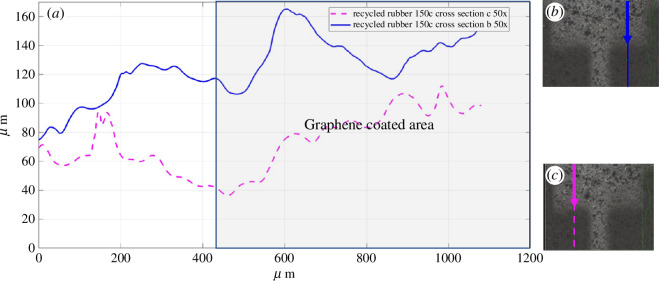
(*a*) Topography of a rubber pad (50% recycled–50% virgin), from uncoated (left-hand side of the plot) to coated with 150 cycles of graphene nanoplatelets (right-hand side of the plot); (*b*) cross-section: blue solid line; (*c*) cross-section: magenta dashed line.

**Figure 5.**
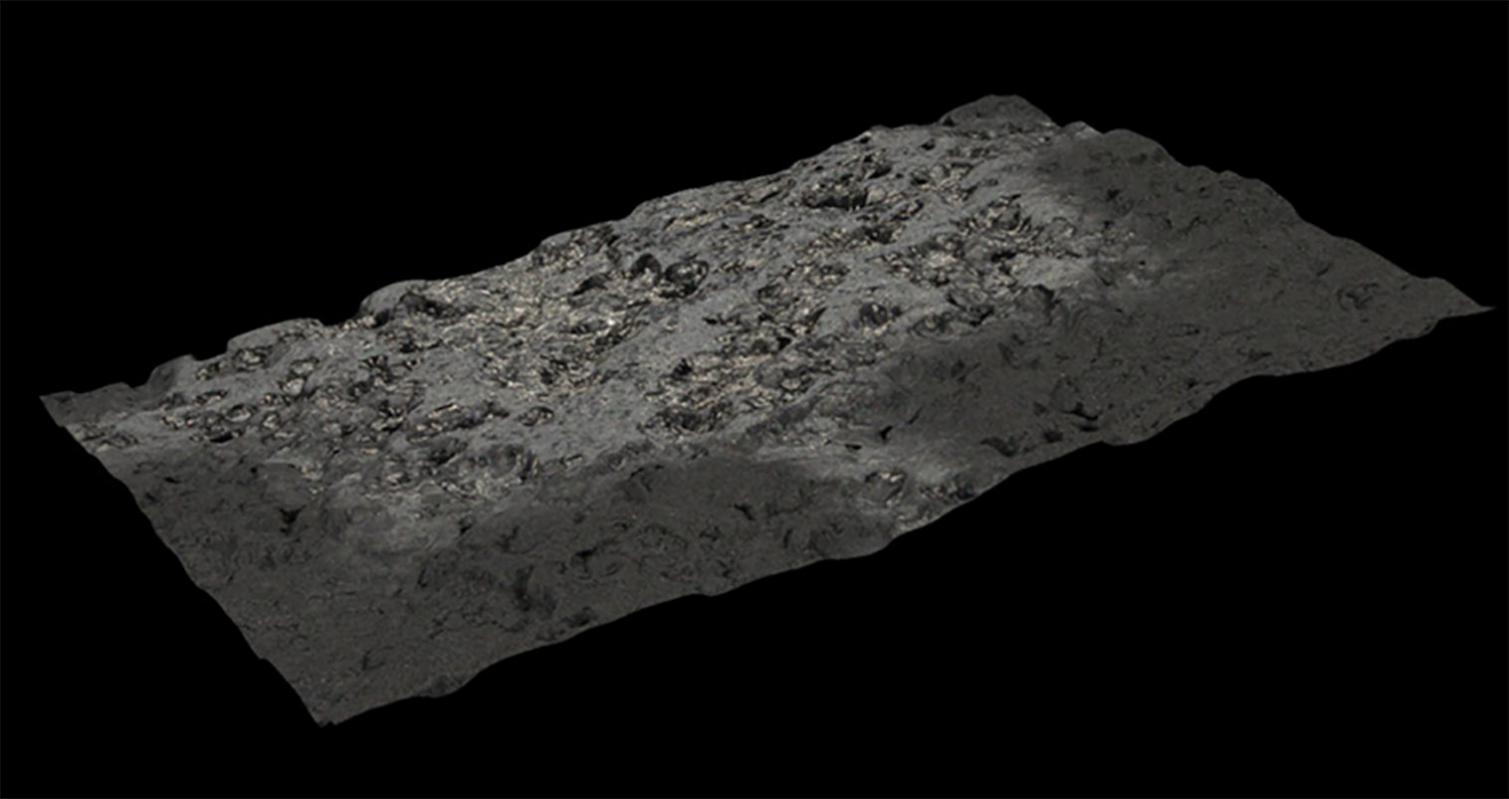
Three-dimensional image of the uneven surface of a rubber pad (50% recycled−50% virgin) coated with 150 cycles of graphene nanoplatelets.

To show the variation in the pad’s thickness when progressing from an uncoated to a coated area, cross-sectional profiles of the pad 2 specimen were generated. Figure 4*a* shows the profile from two cross-sections at 50× magnification from the uncoated (left-hand side of the plot) to the coated area (right-hand side of the plot). The location of the cross-sections is traced in figure 4*b,c* where some graphene nanoplatelets are visible in the uncoated bright areas. Because there are inconsistent rises in the cross-sectional profiles which were only expected to coincide with the presence of graphene covering, the results cannot be considered conclusive. We believe this is due to the high unevenness of the surface of the RR substrate that can be appreciated in the three-dimensional image presented in figure 5.

**Figure 6.**
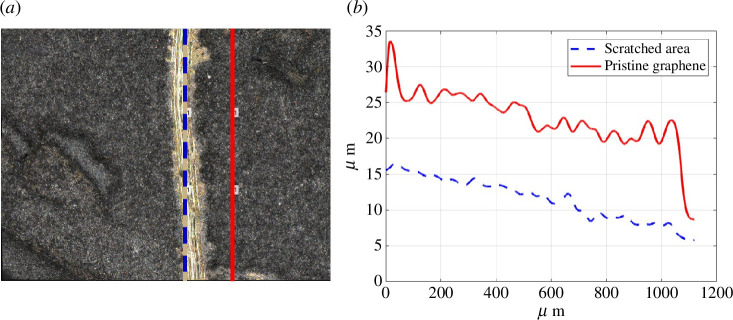
(*a*) Location of scratch on the mask; (*b*) profiles of scratched area (dashed blue) and pristine 150 cumulative cycles graphene nanoplatelets (solid red).

Graphene was inevitably deposited on the mask during the deposition. In order to estimate the thickness of the graphene nanoplatelets’ cumulative cycles, an analysis of a scratch made on the coated mask was carried out, taking advantage of the fact that the surface of the brass mask is more uniform compared with the surface of the rubber. Figure 6*a,b* shows the profiles at 200× magnification traced on the scratched area and on the graphene-coated area used to estimate the thickness of the graphene coating. The gap between the two topographic profiles is around 11 µm, which was estimated to be the thickness of the 150 cumulative cycles of graphene nanoplatelets.

### (d) Scanning electron microscopy

**Figure 7.**
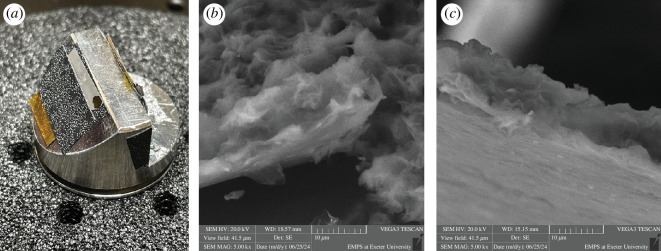
(*a*) Graphene nanoplatelets layer of 150 cumulative cycles on top of SEM specimen holder; (*b,c*) SEM images at 5k× magnification.

To verify the initial estimate of the thickness of 150 cumulative cycles of graphene nanoplatelets performed using a digital microscope, we performed a study at a higher magnification factor using the scanning electron microscopy (SEM) technique. A graphene-coated layer was carefully separated from the mask on which it was deposited and placed on top of the SEM specimen holder (figure 7*a*). SEM images showing the edge of the coated layer at 5k× magnifications factor in two different locations are presented in figure 7*b,c*. It is worth observing that the graphene thickness estimated using the SEM images is around 10 µm; which confirms the estimate from the analysis in §3c.

## 4. Mechanical characterization

To understand the mechanical response of the graphene–RR composite, cyclic compression and shear tests were performed.

### (a) Cyclic compression tests

An Instron Universal Testing Machine with a 30 kN load cell capacity was used to perform compression tests. To accommodate the relaxation of the rubber as well as to ensure contact between the compression platens and the specimen during the tests regardless of its uneven surface, a preload was applied to the specimen before the compression test started and sustained over the duration of the test using a displacement control mode. The specimens in table 2 were compressed up to 4 mm, at the slow rate of 0.05 mm/s for 20 cycles to reduce the influence of rate-dependent effects on the material. The results were analysed to determine the mechanical behaviour of the specimens, namely compressive stiffness and damping.

**Figure 8.**
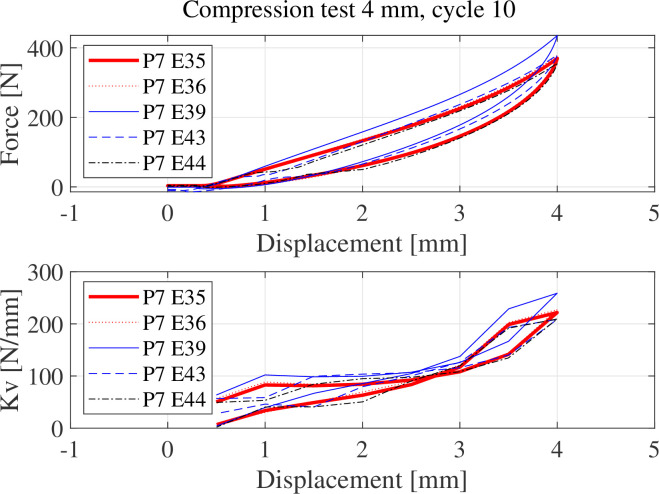
Compressive stiffness *K*_v_ of the first set of specimens at the 10th loading–unloading cycle.

Figure 8 shows the compressive stiffness (*K*_v_) at the 10th compressive loading–unloading cycle of coated (solid line) versus uncoated specimens (dashed lines). The control specimens E43 and E44 (dashed lines) exhibit lower compressive stiffness (*K*_v_ = 209.3 N mm^−1^ at 4 mm) than the specimens made with graphene-coated pads (e.g. E39 *K*_v_ = 258.7 N mm^−1^ at 4 mm), indicating that graphene coating has increased the compressive stiffness of the composite.

**Figure 9.**
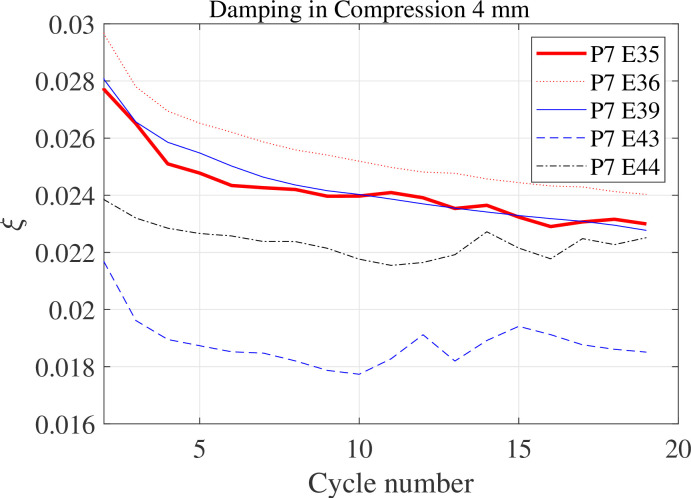
Compressive damping of the first set of specimens for 20 loading–unloading cycles.

Compression damping of the specimens at 4 mm compression for 20 consecutive cycles is shown in figure 9. Damping is characterized as the equivalent viscous damping, which can be determined by measuring the energy dissipated and stored over each loading–unloading cycle using the technique described in Clough [31]. Specifically, the equivalent viscous damping can be evaluated as, ζ = $W_{\text{d}}{\left(2\pi k_{\text{eff}}\Delta_{\text{max}}^{2}\right)}^{-1}$ , where $W_{\text{d}}$ is the energy dissipated in each cycle (which equates the area enclosed by the hysteretic loop), $k_{\text{eff}}$ is the effective compressive stiffness measured as the secant stiffness within the compressive displacement interval 1–3 mm, and $\Delta_{\text{max}}$ is the maximum compressive displacement experienced by the specimens. As can be noticed in figure 8, there are three distinctive regions in the loading path where the compressive stiffness exhibits a linear behaviour with respect to the displacement (i.e. a trilinear model of the hysteretic loop). Thus, the displacement interval used to measure $k_{\text{eff}}$ has been chosen to capture the first of these three representative compressive stiffnesses. The damping estimated from the specimens and presented in figure 9 indicates that the uncoated specimens (E43 and E44, dashed lines) have lower compressive damping than their coated counterparts. This suggests that the presence of graphene nanoparticles interlayers has increased the material compressive damping.

### (b) Cyclic shear tests

**Figure 10.**
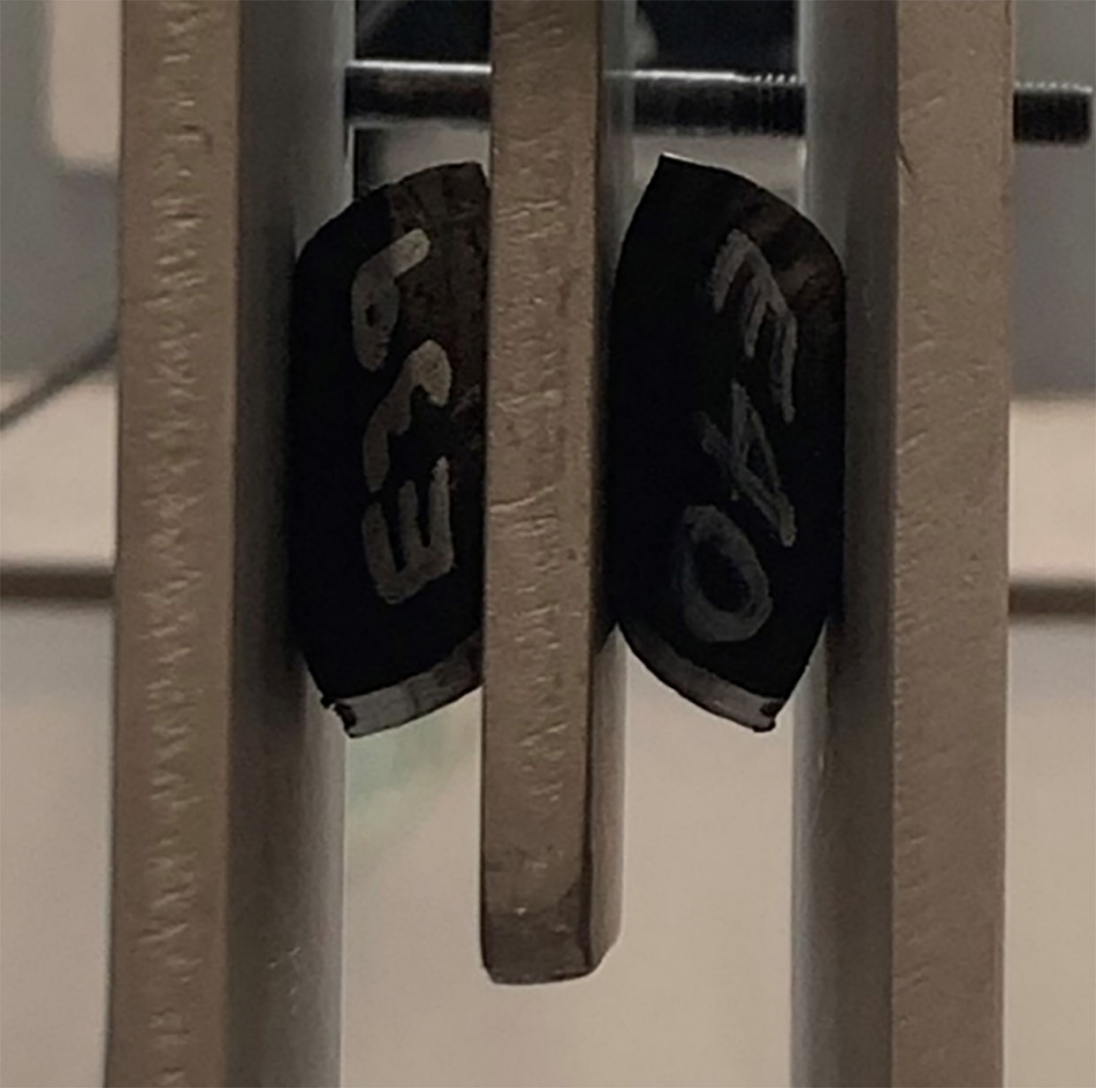
Shear test set up, specimens E39–E40.

The shear tests were carried out using a purpose-built test equipment mounted in an Instron Universal Testing Machine. Specimens were tested in pairs in a symmetrical arrangement. The vertically oriented specimens were positioned between two aluminium outer platens, with an additional central aluminium plate in between (see figure 10). The specimens were subjected to an initial compressive load normal to the platens, which generates frictional contact forces between the specimens and the platens. The central aluminium plate was coupled to the wedge action tensile grips of the Instron machine to simultaneously apply shear displacements to the paired specimens. The machine was operated under displacement control. Shear tests were performed for five cycles at a rate of 0.1 mm s^−1^ and a range of amplitudes corresponding to 20%, 50%, 60% and 70% of the specimen height. It is worth noting that when pulling/pushing the central plate at an amplitude above 50% of the specimen height, the specimens exhibited rollover deformation (i.e. contact surfaces can roll off the support surfaces and no tension stresses are produced in the specimen). This is because the specimens were not mechanically or chemically attached to the outer platens, but instead relied exclusively on friction at their contact surfaces with the outer aluminium platens.

**Figure 11.**
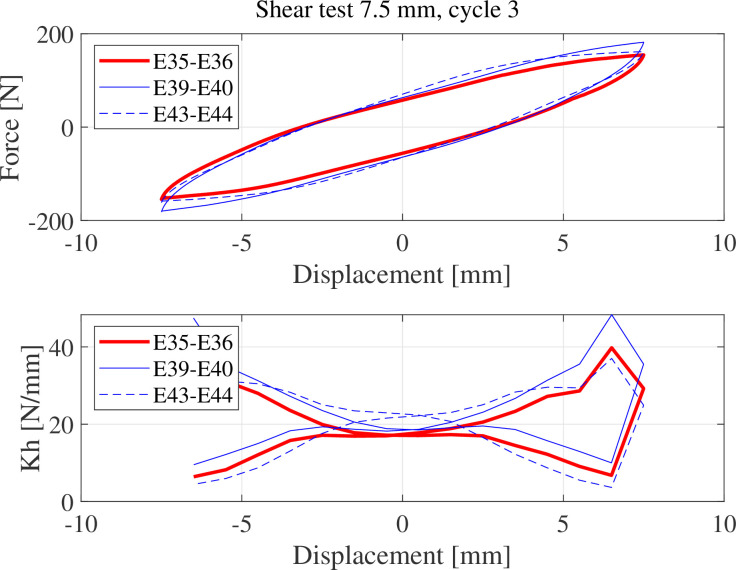
Lateral stiffness *K*_h_ of the paired specimens in the third loading–unloading cycle with 7.5 mm maximum amplitude.

Figure 11 compares the lateral stiffness of three couples of alike specimens in table 2 in the third loading–unloading cycle with 7.5 mm maximum amplitude. Results show that specimens E39–E40 with 40% coated area exhibit reduced lateral stiffness (*K*_h_) as lateral displacement increases. This behaviour is known to be beneficial when the purpose of using the rubber-based composite is to elongate the natural period of a mounted machinery or structure.

## 5. Conclusions

Our study proposes the integration of graphene nanoplatelets to enhance the mechanical properties of RR. Through Raman and FTIR analyses, we investigated the chemical composition of the rubber pads coated with graphene layers. Additionally, the Raman analysis confirmed that the application of a hydrophobic solution on the RR pads employed to improve adhesion during stacking and vulcanization processes has no negative impact on intrinsic rubber structure. FTIR analysis highlighted the potential influence of the exposition to high temperatures during the spray coating process on the material’s elastic properties. Microscopic analysis provided insights into the distribution of graphene nanoplatelets on the uneven rubber substrate and supported attempts to measure the thickness of the graphene layer deposited on it. Several small-scale specimens consisting of stacked coated rubber pads were manufactured and tested under compression and shear loadings. According to preliminary findings, the graphene layers interposed into RR pads enhance compressive stiffness and the corresponding viscous damping while allowing a desirable significant lateral displacement (under shear). Although these findings are promising, further experimental studies are needed to determine the effect of different coating patterns, graphene coverage area and number of graphene coatings on the compressive stiffness and damping of the resulting layered composite.

Further research will investigate the effects of loading rate on the specimen mechanical properties, particularly lateral stiffness and viscous damping in both compression and shear, as well as examining specimen stability. A further research thread will look into developing technologies that allow for repossessing the graphene–RR material at the end of its life in a view to enable circularity. This ongoing investigation promises to advance the understanding of graphene-enhanced recycled rubber for applications for energy absorption and vibration reduction.

## Data Availability

The data that support this paper are available from the corresponding author upon reasonable request.

## References

[1] Millard E . 2019 Recent experiences from the natural rubber industry and its movement towards sustainability. In Sustainable global value chains. natural resource management in transition (eds M Schmidt , D Giovannucci , D Palekhov , B Hansmann ). Cham, Switzerland: Springer. (10.1007/978-3-319-14877-9_27)

[2] Deloitte . 2019 A global state of knowledge on regulation, management systems, impacts of recovery and technologies. World business council for sustainable development. Retrieved from. See https://docs.wbcsd.org/2018/02/TIP/WBCSD_ELT_management_State_of_Knowledge_Report.pdf.

[3] Defra UK: waste and recycling . 2015 Appendix 8 2010 to 2015 government policy: waste and recycling - GOV.UK (www.GOV.UK)

[4] European Recycling Industries’ Confederation -EuRIC . 2022 Mechanical Tyre Recycling Factsheet. See https://euric.org/resource-hub/reports-studies/mechanical-tyre-recycling-factsheet.

[5] Wang JD , Zhu YF , Zhou XW , Sui G , Liang J . 2006 Preparation and mechanical properties of natural rubber powder modified by carbon nanotubes. J. Appl. Polym. Sci. **100** , 4697–4702. (10.1002/app.23076)

[6] Sadasivuni KK , Ponnamma D , Thomas S , Grohens Y . 2014 Evolution from graphite to graphene elastomer composites. Prog. Polym. Sci. **39** , 749–780. (10.1016/j.progpolymsci.2013.08.003)

[7] Papageorgiou DG , Kinloch IA , Young RJ . 2015 Graphene/elastomer nanocomposites. Carbon **95** , 460–484. (10.1016/j.carbon.2015.08.055)

[8] Young RJ , Liu M , Kinloch IA , Li S , Zhao X , Vallés C , Papageorgiou DG . 2018 The mechanics of reinforcement of polymers by graphene nanoplatelets. Compos. Sci. Technol. **154** , 110–116. (10.1016/j.compscitech.2017.11.007)

[9] Papageorgiou DG , Kinloch IA , Young RJ . 2017 Mechanical properties of graphene and graphene-based nanocomposites. Prog. Mater. Sci. **90** , 75–127. (10.1016/j.pmatsci.2017.07.004)

[10] Mensah B , Gupta KC , Kim H , Wang W , Jeong KU , Nah C . 2018 Graphene-reinforced elastomeric nanocomposites: a review. Polym. Test. **68** , 160–184. (10.1016/j.polymertesting.2018.04.009)

[11] Zhang H , Xing W , Li H , Xie Z , Huang G , Wu J . 2019 Fundamental researches on graphene/rubber nanocomposites. Adv. Ind. Eng. Polym. Res. **2** , 32–41. (10.1016/j.aiepr.2019.01.001)

[12] Lim LP , Juan JC , Huang NM , Goh LK , Leng FP , Loh YY . 2020 Effect of graphene oxide particle size on the tensile strength and stability of natural rubber graphene composite. Mat. Sci. Eng.: B **262** , 114762. (10.1016/j.mseb.2020.114762)

[13] Sayfo P , Pirityi DZ , Pölöskei K . 2023 Characterization of graphene-rubber nanocomposites: a review. Mat. Today Chem. **29** , 101397. (10.1016/j.mtchem.2023.101397)

[14] Innes JR , Young RJ , Papageorgiou DG . 2022 Graphene nanoplatelets as a replacement for carbon black in rubber compounds. Polymers **14** , 1204. (10.3390/polym14061204) 35335535 PMC8949821

[15] Wu J , Xing W , Huang G , Li H , Tang M , Wu S , Liu Y . 2013 Vulcanization kinetics of graphene/natural rubber nanocomposites. Polymer **54** , 3314–3323. (10.1016/j.polymer.2013.04.044)

[16] Lim LP , Juan JC , Huang NM , Goh LK , Leng FP , Loh YY . 2019 Enhanced tensile strength and thermal conductivity of natural rubber graphene composite properties via rubber-graphene interaction. Mater. Sci. Eng. B **246** , 112–119. (10.1016/j.mseb.2019.06.004)

[17] Jibin KP , Prajitha V , Thomas S . 2021 Silica-graphene oxide reinforced rubber composites. Mat. Today. **34** , 502–505. (10.1016/j.matpr.2020.03.100)

[18] Sun X , Huang C , Wang L , Liang L , Cheng Y , Fei W , Li Y . 2021 Recent progress in graphene/polymer nanocomposites. Adv. Mater. Weinheim **33** . (10.1002/adma.202001105) 32893409

[19] Sabet M . 2024 Unveiling transformative potential: recent advances in graphene-based polymer composites. Iran. Polym. J. (10.1007/s13726-024-01337-2)

[20] Fu X *et al* . 2023 Graphene oxide as a promising nanofiller for polymer composite. Surf. Interf. **37** , 102747. (10.1016/j.surfin.2023.102747)

[21] Asadollahi-yazdi H , Shariati M , Imam A , Ghatee M . 2017 Investigating the mechanical properties of layered graphene/polyoxymethylene nanocomposites prepared by the spray method. J. Compos. Mater. **51** , 3053–3064. (10.1177/0021998316681188)

[22] Marsico MR , Londoño Monsalve JM , Lu L , Craciun MF . 2022 The effect of graphene ultrasonic coating on recycled rubber. Adv. Eng. Mater. **24** , 2200957. (10.1002/adem.202200957)

[23] Stryckers J , Swusten T , Brullot W , D’Haen J , Verbiest T , Deferme W . 2018 Ultrasonic spray coating as a fast alternative technique for the deposition of hybrid magnetic‐plasmonic nanocomposites. Adv. Eng. Mater. **20** , 1800681. (10.1002/adem.201800681)

[24] Marsico MR , Londoño Monsalve JM , Shin DW , Craciun MF . 2020 Graphene–rubber layered functional composites for seismic isolation of structures. Adv. Eng. Mater. **22** , 1900852. (10.1002/adem.201900852)

[25] An S , Joshi B , Yarin AL , Swihart MT , Yoon SS . 2020 Supersonic cold spraying for energy and environmental applications: one-step scalable coating technology for advanced micro- and nanotextured materials. Adv. Mater. Weinheim **32** , e1905028. (10.1002/adma.201905028) PMC6980375 31747097

[26] Sadanandan KS , Bacon A , Shin DW , Alkhalifa S , Russo S , Craciun MF , Neves A . 2020 Graphene coated fabrics by ultrasonic spray coating for wearable electronics and smart textiles. J. Phys. Mater. **4** , 014004. (10.1088/2515-7639/abc632)

[27] Ferrari AC *et al* . 2006 Raman spectrum of graphene and graphene layers. Phys. Rev. Lett. **97** , 187401. (10.1103/PhysRevLett.97.187401) 17155573

[28] Dresselhaus MS , Jorio A , Hofmann M , Dresselhaus G , Saito R . 2010 Perspectives on carbon nanotubes and graphene raman spectroscopy. Nano Lett. **10** , 751–758. (10.1021/nl904286r) 20085345

[29] Ţucureanu V , Matei A , Avram AM . 2016 FTIR spectroscopy for carbon family study. Crit. Rev. Anal. Chem. **46** , 502–520. (10.1080/10408347.2016.1157013) 26941009

[30] Gunasekaran S , Natarajan RK , Kala A . 2007 FTIR spectra and mechanical strength analysis of some selected rubber derivatives. Spectrochim. Acta A Mol. Biomol. Spectrosc. **68** , 323–330. (10.1016/j.saa.2006.11.039) 17320472

[31] Clough R , Penzien J . 1975 Dynamics of structures. New York, NY: McGrow-Hill.

